# Sociodemographic and Reproductive Risk Factors for Breast Cancer: A Case-Control Study in the Setif Province, Northern Algeria

**DOI:** 10.31557/apjcp.2020.21.2.457

**Published:** 2020

**Authors:** Mokhtar Hamdi-Cherif, Diego Serraino, Souad Bouaoud, Adlane Dib, Khadidja Boudaoud, Saida Atoui, Imene Merghem, Federica Toffolutti, Ettore Bidoli, Lamia Kara, Asma Ayat, Ghania Habia, Kenza Makhloufi, Imane Bouchaibi, Sabah Kettaf, Samiha Chenafi, Douniazad Dilmi, Karima Bouhafs, Bahadinne Ablaoui, Hadjira Chaouche, Loubna Belbedj, Asma Nadjem, Nawel Lakab, Saverio Virdone, Chiara Panato

**Affiliations:** 1 *Faculty of Medicine and Cancer Registry, University of Setif, Setif, Algeria, *; 2 *Cancer Epidemiology Unit, Oncological Reference Center (CRO), IRCCS, *; 3 *Friuli Venezia Giulia Cancer Registry, IRCCS Oncological Reference Center, Aviano, Italy. *

**Keywords:** Breast cancer, reproductive habits, family history, case-control study

## Abstract

**Background:**

The age-standardized rate of breast cancer (BC) increased nearly nine-fold in the last 30 years in Setif, Algeria. A case-control study was carried out to investigate the role of sociodemographic characteristics and reproductive factors in the etiology of BC in this young Arab/Berber population of Setif.

**Methods:**

Cases were 612 women with incident BC admitted to major teaching and general hospitals of Setif during 2012–2017. Controls were 615 women admitted for acute, non-neoplastic conditions to the same hospital network of cases. Information was elicited using a standardized questionnaire. Odds ratios (ORs) and 95% confidence intervals (CIs) were computed after allowance for age and education.

**Results:**

Significant associations with BC risk emerged for family history of BC (OR= 4.15 for yes vs. no; 95%CI: 2.22-7.77), for the generation of oral contraceptive used (OR=1.57 for II-generation vs. III-generation; 95%CI:1.01-2.44), and education (OR=0.63 for >11 years vs. no schooling; 95%CI: 0.46-0.86). Conversely, parity and body mass index were not associated with BC risk, whereas a late age at menarche was linked with a non statistically significant BC risk (OR=1.20 for >15 vs. <13 years; 95%CI:0.86-1.66). These results were consistent in strata of menopausal status.

**Conclusion(s):**

Some of the expected risk factors (e.g., low education levels and type of oral contraceptives) were associated with elevated BC risks also in Setif, and these findings offer further support to primary preventive efforts already carried on in Algeria. Conversely, no association emerged for other well established risk factors (e.g., body mass index).

## Introduction

Breast cancer (BC) is the most frequent cancer diagnosed in women worldwide (Ferlay et al., 2013). In the province of Setif, northern Algeria, BC accounts for 44.5% of all incident female cancers, excluding non-melanoma skin cancers (Association ENNOUR, 2019). The age-standardized rate (world population) of BC was 55.4/100,000 in 2016, an increase of about nine-fold since 1986 (Association ENNOUR, 2019; Hamdi Cherif et al., 2014). Although this incidence rate is still 30% to 50% lower than the rates recorded by the European cancer registries located in the northern Mediterranean rim (Bray et al., 2017), the BC picture is rapidly worsening (Zanetti et al., 2010; Corbex et al., 2014), in North Africa. 

Several modifiable and non-modifiable risk factors are associated with BC. Chiefly, the menstrual and reproductive characteristics that increase the risk of BC are linked both to prolonged endogenous oestrogen exposure (i.e., to early menarche, low parity, late childbearing, shorter period of breastfeeding, or late menopause) (Collaborative Group on Hormonal Factors in Breast Cancer, 2012), and exogenous oestrogen exposure (i.e., use of oral contraceptive pills or hormone replacement therapy) (Collaborative Group on Hormonal Factors in Breast Cancer, 1996). Finally, a family history of BC is one of the strongest and consistent risk factor for BC (Collaborative Group on Hormonal Factors in Breast Cancer, 2001). 

In the Setif context (1.2 million inhabitants), the mean age at BC diagnosis was around 45 years (Association ENNOUR, 2019) a likely consequence of the Setif population pyramid that is skewed toward younger age groups (Office National des Statistiques, 2018). However, the exact role of the well-established risk factors in industrialized countries is not well documented in the young Algerian women. Therefore, it is not clear to what extent the observed increasing time trends of BC incidence could be attributed to already established risk factors, and/or to differences in the magnitude of their association with BC.

The aim of this case-control study was to explore the relationship between socio-economic and reproductive characteristics and BC risk in Algerian women residing in the Setif province. To the best of our knowledge, this is the first epidemiological study on BC risk factors conducted in Algeria.

## Materials and Methods

A case-control study on BC was carried out between September 2012 and December 2017. Cases were 612 women (median age 45 years, range 28–77 years) with incident, histologically confirmed BC less than one year prior to interview (median 6 months) with no previous diagnosis of any cancer type. Controls were 615 women frequency matched to cases with a 1:1 ratio on age (median age 46 years, range 27–79 years) admitted to the same network of hospitals of cases for a wide spectrum of non-neoplastic acute illnesses. These control women were admitted for traumatic orthopedic disorders (mainly broken harms, hands, and legs), other orthopedic disorders, acute surgical conditions (mainly appendicitis and hernias), and other miscellaneous illnesses, including eye, nose, ear, skin or dental disorders. Women hospitalized for medical conditions associated with long-term dietary changes were not eligible. Overall, less than 5% of approached cases and controls refused to be interviewed. 

The approval of a research ethic committee is not necessary in Algeria for an observational study since no intervention on patients took place in this study. Nonetheless, the study received clearance by the Internal Review Board of the Ferhat-Abbas University, Setif. Although a signed informed consent is not required by Algerian law, all eligible patients had given their oral consent to take part into the study before conducting the interviews.

A structured questionnaire was administered by centrally trained interviewers during the patients’ hospital stay, including information on: personal characteristics and lifestyle habits; anthropometric measures; a problem-oriented medical history; a history of any cancer in first-degree relatives (i.e., parents, siblings, and offspring); menstrual and reproductive characteristics; use of oral contraceptive (OC); and hormone replacement therapy (HRT). The questionnaire was a French translation of an existing Italian questionnaire used in our case-control studies, which had been previously tested for validity and reproducibility (Decarli et al., 1996; Franceschi et al., 1995). Two women declared to have used HRT. Alcohol consumption was not investigated since it is not consumed in Algeria, due to religion issues and restrictions. The body mass index (BMI) was calculated using the standard formula, i.e., weight in kg/height in m^2^, while the estimated reproductive interval was calculated as age at menopause minus age at menarche, minus one year for each full-term pregnancy.

The questionnaires contained several general questions about OC use (ever/never), age at first use, total duration, and brand name of the OCs. We used the generation of the product to classify OCs, which was based on the time of their marketing (Haute Autorité de Santé, 2019) and consequently on the concentrations of estrogens and progestins contents. The second-generation pills began to be manufactured from 1970s onward, and the third-generation came on the market in the 1980s. 

Odds Ratios (OR) and their corresponding 95% confidence intervals (CI) were computed using unconditional multiple logistic regression models (Breslow and Day 1980) adjusted for age in quinquennia and education (illiterate, 1-5, 6-10, and >11 school years). All statistical analyses were carried out with SAS 9.4 statistical software (SAS Institute 2019). The sample size of our study (612 cases and 615 controls, i.e a case:control ratio of 1) had a power of 80% to detect an OR of 1.4 when the percentage of controls “exposed” was 25% (considering also a type I error of 5%, with two-tails). To give clues on the association between studied variables and breast cancer, in the Setif population, we reported the ORs (i.e., the maximum likelihood estimates of the risk) even when, in some cases, the power was lower than 80%. 

## Results

Table 1 gives the distribution of BC cases (n=612) and controls (n=615) according to age, education, marital status, and BMI, together with multivariate (adjusted for age and education) ORs of BC. By design, cases and controls had similar age distributions. Controls tended to be more educated than cases (X^2^_3_=8.45; p=0.04), and increasing years of education were inversely related to the risk of BC (in particular, OR=0.63 for ≥11 years vs. no schooling; 95%CI: 0.46-0.86). One-hundred seventy-eight cases (29%) and 196 controls (31%) displayed a BMI ≥30 kg/m^2^. No statistically significant difference between cases and controls was observed for BMI (OR=1.04 for BMI≥32 vs. BMI<25; 95%CI:0.74-1.45). Married and divorced/widowed women displayed a slight excess of risk, though such difference was not statistically significant (OR=1.29 for divorced/widow vs. never married, 95%CI:0.79-2.09, and OR=1.14 for married vs. never married, 95%CI: 0.79-1.62).

Table 2 describes the distribution of BC cases and controls according to selected menstrual and reproductive characteristics (i.e., age at menarche, menses frequency, parity, type of abortion, oral contraceptive use and the generation of oral contraceptives pills mainly used, menopause status, age at menopause, type of menopause, estimated reproductive interval, and family history of BC) together with multivariate ORs of BC. A direct statistically significant association with BC emerged for menopausal status (OR=2.92 for post- vs. pre-menopause; 95%CI:1.98-4.29), type of menopause (OR=7.31 for artificial vs. natural menopause; 95%CI:3.14-17.01), family history of BC (OR= 4.15 for yes vs. no; 95%CI: 2.22-7.77). 

Three-hundred forty-five cases (63%) and 321controls (59%) ever used OCs. A difference of borderline statistical significance was noted for use of any OCs on BC risk (OR=1.24 for ever vs. never; 95%CI:0.96-1.60). However, when the use of OCs was examined separately by the generation of the pills (second, third, and a mixture of pills generations), a direct risk emerged for women with lifelong use of the second-generation pills vs. the third-generation lifelong use (OR=1.57; 95%CI:1.01-2.44). A direct -though not statistically significant- risk emerged for women who swapped back and forth from second- to third-generation pills vs. those who used only third-generation lifelong (OR=1.56; 95%CI:0.88-2.75); whereas, an inverse BC risk was observed for the seven women (two BC cases and five controls) who used a mixture of second-, third-, and fourth- generation pills (OR=0.46; 95%CI:0.09-2.50). None of the women enrolled in the study declared to have used first-generation pills, while seven women (two cases and five controls) declared to have used fourth-generation pills, alone or in combination with second-generation pills.

An elevated number of spontaneous abortions –83 cases and 62 controls had at least 2 spontaneous abortions– was directly related to BC (OR=1.42 for ≥2 vs. 0 abortions; 95%CI:0.99-2.04), whereas the number of induced abortions –11 cases and 12 controls had any induced abortions– and the age at any first abortion were unrelated to BC risk. Spontaneous abortions (never vs. ever) stratified by parity (nulliparous vs. parous) were further examined. For nulliparous women OR=1.91(95%CI:0.51-6.65) for spontaneous abortions vs. never. In parous women OR=1.26 (95%CI:0.96-1.64) for spontaneous abortions vs. never. Several direct associations with BC risk, though not statistically significant, were also observed for age at menarche (OR=1.20 for ≥15 vs. <13 years; 95%CI:0.86-1.66), menses frequency (OR=1.30 for ≥31 vs. 26-30 days; 95%CI:0.83-2.04), age at first marriage (OR=1.36 for >30 vs. <20 years; 95%CI:0.91-2.04), and estimated reproductive life (OR=1.19 for ≥30 vs. <30 years; 95%CI: 0.83-1.72).

The relationship between the selected reproductive characteristics and BC risk was further examined in two strata of menopausal status (pre-/peri-menopause, and post-menopause) (data not shown). Although some differences in the estimated risks were observed across strata, these were compatible with the effect of random variation, since none of the heterogeneity tests were significant. 

**Table 1:** Distribution of 612 Cases of Breast Cancer and 615 Controls and Odds Ratios (OR)* of breast Cancer and Corresponding 95% confidence intervals (CI) according to selected variables†. Setif, Algeria. 2012-2016

	Cases		Controls		OR	(95% CI)
Variable	N	(%)	N	(%)		
Age (years)						
<40	163	(26.6)	151	(24.6)	-	
40-44	135	(22.1)	133	(21.6)	-	
45-49	104	(17.0)	105	(17.1)	-	
50-59	141	(23)	155	(25.2)	-	
≥60	69	(11.3)	71	(11.5)	-	
Median age of patients	45	-	46	-	-	
*X* ^2^_4_ *=1.16; p-value=0.88*						
Education (years)						
0	217	(36.2)	170	(28.4)	1	
1-5	54	(9.0)	62	(10.4)	0.68	(0.45-1.03)
6-10	180	(30.0)	196	(32.7)	0.68	(0.51-0.92)
≥11	149	(24.8)	171	(28.6)	0.63	(0.46-0.86)
*X* ^2^_3_ *=8.45; p-value=0.04*						
Marital status						
Never married	73	(12.0)	74	(12.3)	1	
Divorced/widow	75	(12.3)	68	(11.3)	1.29	(0.79-2.09)
Married	460	(75.7)	462	(76.5)	1.14	(0.79-1.62)
*X* ^2^_2_ *=0.34; p-value=0.84*						
Body Mass Index (kg/m^2^)						
<25	143	(25.7)	143	(25.5)	1	
25-29	236	(42.4)	225	(40.2)	1.09	(0.83-1.43)
30-31	70	(12.6)	85	(15.2)	0.86	(0.59-1.25)
≥32	108	(19.4)	107	(19.1)	1.04	(0.74-1.45)
*X* ^2^_3_ *=1.71; p-value=0.63*						

*estimates adjusted for age (quinquennia) and education (when appropriate); †, the sums may not add up to the total because of missing values.

**Table 2:** Odds Ratios (OR)* of Breast Cancer and Corresponding 95% Confidence Intervals (CI) According to Selected Menstrual and Reproductive Variables†. Setif, Algeria. 2012-2016

Variable	Cases		Controls		OR	(95% CI)
	N	(%)	N	(%)		
Age at menarche (years)						
<13	213	(35.6)	225	(37.3)	1	
13-14	271	(45.2)	276	(45.7)	1.06	(0.82-1.37)
≥15	115	(19.2)	103	(17.1)	1.2	(0.86-1.66)
Menses frequency (days)						
26-30	447	(75.4)	460	(77.6)	1	
<26	99	(16.7)	95	(16.0)	1.11	(0.82-1.52)
≥31	47	(7.9)	38	(6.4)	1.30	(0.83-2.04)
Age at first marriage (years)						
<20	190	(36.0)	204	(37.5)	1	
20-24	144	(27.3)	160	(29.4)	1.01	(0.74-1.39)
25-29	118	(22.4)	117	(21.5)	1.12	(0.79-1.60)
≥30	76	(14.4)	63	(11.6)	1.36	(0.91-2.04)
Age at first birth (years)						
13-21	165	(37.0)	175	(37.7)	1	
22-26	138	(30.9)	141	(30.4)	1.06	(0.76-1.48)
≥27	143	(32.1)	148	(31.9)	1.04	(0.74-1.46)
Parity						
Nulliparous	106	(17.9)	86	(14.5)	1	
1-2	112	(18.9)	105	(17.7)	0.96	(0.66-1.40)
3-5	252	(42.5)	292	(49.1)	0.79	(0.58-1.08)
≥6	123	(20.7)	112	(18.8)	0.97	(0.66-1.44)
Number of spontaneous abortions						
0	375	(65.7)	399	(70.3)	1	
1	113	(18.8)	107	(18.8)	1.13	(0.84-1.53)
≥2	83	(14.5)	62	(10.9)	1.42	(0.99-2.04)
Number of induced abortions						
0	527	(98.0)	513	(97.7)	1	
1	7	(1.3)	7	(1.3)	0.98	(0.34-2.83)
≥2	4	(0.7)	5	(1.0)	0.85	(0.22-3.21)
Age at any first abortion (years)						
13-29	87	(55.1)	89	(57.8)	1	
≥30	71	(44.9)	65	(42.2)	1.19	(0.75-1.88)
Any oral contraceptive use						
Never	202	(36.9)	222	(40.9)	1	
Ever	345	(63.1)	321	(59.1)	1.24	(0.96-1.60)
Oral Contraceptive generation pill						
II lifelong	151	(59.2)	138	(54.3)	1.57	(1.01-2.44)
III lifelong	59	(23.1)	75	(29.5)	1	
II and /or III	43	(16.9)	36	(14.2)	1.56	(0.88-2.75)
II and/or IV‡	2	(0.8)	5	(2.0)	0.46	(0.09-2.50)
Menopausal status						
Pre-	192	(34.1)	241	(42.5)	1	
Peri-	76	(13.5)	82	(14.5)	1.37	(0.94-2.02)
Post-	295	(52.4)	244	(43.0)	2.92	(1.98-4.29)
Age at menopause (years)						
<46	135	(48.4)	83	(35.9)	1	
≥46	144	(51.6)	148	(64.1)	0.75	(0.51-1.11)
Type of menopause						
Natural	228	(77.3)	232	(96.7)	1	
Artificial	67	(22.7)	8	(3.3)	7.31	(3.14-17.01)
Estimated reproductive life§ (years)						
<30	167	(56.7)	133	(54.5)	1	
≥30	128	(43.4)	111	(45.5)	1.19	(0.83-1.72)
Family history of breast cancer						
No	564	(92.2)	602	(97.9)	1	
Yes	48	(7.8)	13	(2.1)	4.15	(2.22-7.77)

*, estimates adjusted for age (quinquennia) and education; †, the sum may not add up to the total because of missing values; ‡, 4 patients with II+IV OC generation, and 3 patients with IV OC generation; §, calculated as: age at menopause minus age at menarche minus one year for each full-term pregnancy.

## Discussion

Our study, one of the largest case-control investigations on BC risk to date in a north African population consisting mainly of young women, showed that a family history of BC, the use of second-generation OCs and being in post-menopause were directly associated with the risk of developing BC, while a higher education was inversely related to BC risk. Moreover, our study showed a direct, though not significant, association of age at menarche with BC risk but no protective association of parity or no detrimental effect of BMI. 

These results are mainly consistent with findings from other epidemiological investigations conducted both in industrialized countries and in Arab countries. Several biological plausible mechanisms linked to female hormones may explain several of the observed associations.

In this case-control study, we observed that a higher education level was inversely associated with BC risk as in some studies (Balekouzou et al., 2017; Goldberg et al., 2015), whereas illiterates or low educated women were at lower risk of BC in other studies (Hosseinzadeh et al., 2014; Cunningham et al., 2010). The risk of BC tended to increase among married or divorced/widowed women as compared to never married women, as in previous studies (Balekouzou et al., 2017; Bhadoria et al., 2013). It is thus possible that higher education or never married women may have more opportunities of gaining awareness of BC risk factors and practice a healthier lifestyle. 

In industrialized countries, studies that focused on pre-menopausal women showed that late age at menarche was associated with BC risk (Elkum et al., 2014; Sighoko et al., 2013; Ronco et al., 2012). By contrast, our results indicated that both pre- and post-menopausal women were at higher risk of BC with regard to late age at menarche. Generally, the literature reports that a longer exposure to estrogens, i.e., early menarche women (Collaborative Group on Hormonal Factors in Breast Cancer, 2012), is a BC risk factor. Nonetheless, we observed that a higher reproductive interval tended to be associated with BC risk, supporting the detrimental role of a longer exposure to estrogens (Collaborative Group on Hormonal Factors in Breast Cancer, 2012).

A family history of BC was highly associated with BC also among these study participants in Setif as already described worldwide (Collaborative Group on Hormonal Factors in Breast Cancer, 2001). Our observation is in line with a study that measured a total of 20% of BRCA1 or BRCA2 deleterious germline mutations (Henouda et al., 2016) on forty Algerian primary invasive unrelated BC cases who attended the Anti-Cancer Center of Setif. In addition, a second study conducted on an Algerian cohort of 70 families with a personal and family history of BC found the presence of a large BRCA1 and BRCA2 mutation spectrum in North African populations (Cherbal et al., 2012). 

An increased parity was not associated with a decreased risk of BC, as observed in other population groups (Nindrea et al., 2017). The same puzzling pattern was observed for age at first birth (i.e., no protection was conferred by an early age at first full-term child delivery) (Albrektsen et al., 1994). This pattern may indicate the effect of a transient increase in risk of BC following an elevated multi-parity. Considering that women were diagnosed at a young age (i.e., the mean age was 45 years) and that the increase in risk following a pregnancy runs around 3-4 years, it is merely possible that the parity-induced protection in BC risk was attenuated in this population (Albrektsen et al., 1994; Mathelin et al., 2007).

Worldwide, a positive association between abortion and BC has been frequently reported by case-control studies (Balekouzou et al., 2017; Hosseinzadeh et al., 2014), but not by prospective studies (Clavel-Chapelon, et al., 2002; Guo et al., 2015). Findings from this case-control study highlighted an association with the number of spontaneous abortions, but not with induced abortions, in line with other similarly designed studies. Irregular menstrual cycles are known to represent a risk factor for BC, an association still debated and possibly related to variations in exposure to estrogens throughout life (Henderson et al., 1988). 

No association was observed between BC and BMI, both in pre- or post-menopausal women. Obesity is linked to BC risk in postmenopausal, BC particularly in women not using HRT. By contrast, obesity is associated with reduced premenopausal BC risk. These associations are hormone receptor-positive dependent (i.e., estrogen receptor [ER]-positive and/or progesterone receptor–positive tumors) (Bandera and John, 2018; World Cancer Fund International/American Institute for Cancer Research, 2017), a pattern to be further clarified in the female population of Setif.

The observed borderline significance association with the use of any OC is in line with what expected (Collaborative Group on Hormonal Factors in Breast Cancer, 1996). The lifelong exclusive use of second-generation pills was associated with BC risk as already seen in a Norwegian cohort study (Dumeaux et al., 2003), whereas in a study conducted on US women aged 35-64 years, no evidence of risk was detected according to pill formulation (Marchbanks et al., 2012).

Given the retrospective nature of our study, recall bias is a possible weakness. However, awareness of any particular menstrual or reproductive characteristics on BC etiology had not received media attention in Algeria at the time of the study. Although cases might have reported their lifestyle habits differently than controls, this bias should be very limited in consideration of the observed results, which are mainly in line with international studies. Moreover, the comparability of recall between cases and controls was improved by interviewing all subjects in a hospital setting. With reference to selection bias, the catchment areas were comparable for cases and controls. Cases enrolled in the present study represented a proportion of all BCs arisen in the Setif area during the study period, but their age distribution was similar. 

Conversely, the study suffered from two substantial limitations, i.e., the impossibility of measuring the waist-to-hip ratio, due to denial of the patients; and lack of knowledge on ages at beginning and end of consumption of the various OCs in different periods of life. Moreover, estrogen-receptors and progesterone-receptors that could help in the interpretation of results were not available. Finally, our findings on BMI may be related to weight loss associated with advanced breast cancer, although we elicited the weight two years before diagnosis. Our findings were strengthened by the relatively large dataset used, the nearly complete participation of identified cases and controls, the use of a standardized questionnaire, and the assessment of a broad range of menstrual and reproductive characteristics, which increased detection of any meaningful association. A large proportion of cases were detected because of clinical symptoms, thus reducing the possibility of detection bias. Although, allowance was made for the two observed potential study confounding factors, hidden confounding may still be present. Additional allowance for menopausal status did not meaningfully change the results. 

In conclusion, this study showed the presence of well-known risk factors of BC, and also a pattern of risk peculiar to this north African population of mainly young women. These risk factors are the result of lifestyle habits, which are modifiable, and of genetic unmodifiable factors, whose consequences can be lessened. Thereby, these findings could be of great value to establish adequate evidence-based awareness and BC preventive measures in Algerian women. 

## Data Availability

The datasets used and/or analyzed during the current study are available from the first author (MHC) on reasonable request.
